# The Mayo conservative hip: complication analysis and management of the first 41 cases performed at a University level 1 department

**DOI:** 10.1186/s12891-017-1613-2

**Published:** 2017-06-09

**Authors:** Jörg Arnholdt, Fabian Gilbert, Marc Blank, Jannis Papazoglou, Maximilian Rudert, Ulrich Nöth, Andre F. Steinert

**Affiliations:** 1 Department of Orthopaedic Surgery, König-Ludwig-Haus, Julius-Maximilians-University, Brettreichstr. 11, 97074 Würzburg, Germany; 2 Department of Trauma, Hand, Plastic and Reconstructive Surgery, University Hospital Wuerzburg, Oberduerrbacher Str. 6, 97080 Würzburg, Germany; 3 Department of Orthopaedic Surgery and Traumatology, Evangelisches Waldkrankenhaus Spandau, Stadtrandstraße 555, 13589 Berlin, Germany; 4 Department of Trauma and Orthopaedic Surgery, Agatharied Hospital, Norbert-Kerkel-Platz, 83734 Hausham, Germany

**Keywords:** Total hip arthroplasty, Short hip stem, Mayo stem, Minimal invasive surgery

## Abstract

**Background:**

To prevent bone loss in hip arthroplasty, several short stem systems have been developed, including the Mayo conservative hip system. While there is a plethora of data confirming inherent advantages of these systems, only little is known about potential complications, especially when surgeons start to use these systems.

**Methods:**

In this study, we present a retrospective analysis of the patients’ outcome, complications and the complication management of the first 41 Mayo conservative hips performed in 37 patients. For this reason, functional scores, radiographic analyses, peri- and postoperative complications were assessed at an average follow-up of 35 months.

**Results:**

The overall HHS improved from 61.2 pre-operatively to 85.6 post-operatively. The German Extra Short Musculoskeletal Function Assessment Questionnaire (XSFMA-D) improved from 30.3 pre-operatively to 12.2 post-operatively. The most common complication was an intraoperative non-displaced fracture of the proximal femur observed in 5 cases (12.1%). Diabetes, higher BMI and older ages were shown to be risk factors for these intra-operative periprosthetic fractures (*p* < 0.01). Radiographic analysis revealed a good offset reconstruction in all cases.

**Conclusion:**

In our series, a high complication rate with 12.1% of non-displaced proximal femoral fractures was observed using the Mayo conservative hip. This may be attributed to the flat learning curve of the system or the inherent patient characteristics of the presented cohort.

## Background

Total hip arthroplasty (THA) is one of the most effective procedures in orthopaedic surgery. It provides pain relief and restores function as well as mobility. However, the optimal management of young individuals with osteoarthritis (OA) of the hip is still the subject of scientific controversy [1, 2] . The principle of minimally invasive hip surgery (MIS) includes soft-tissue sparing techniques and conservation of autochthonous bone stock. It has been demonstrated that minimally invasive surgical approaches to the hip, e.g. the minimally-invasive direct anterior approach, might be able to reduce postoperative pain levels, decrease the intake of pain medication and shorten hospitalisation [3]. Soft-tissue preservation and conservation of authochthonous bone stock remains a major challenge in THA.

A steadily increasing number of young patients undergo total hip arthroplasty as quality of life standards in Western societies have increased during the last century while patients demand a great deal of their mobility even at higher ages [1, 2]. Younger individuals are more likely in need of revision surgery with adequate femoral bone stock being essential for satisfactory results following revision arthroplasty. Resurfacing implants have shown acceptable short- and midterm results compared to conventional designs but higher complication rates such as fractures of the femoral neck and generation of metal ions that can be detected systemically [4, 5]. Short stem systems offer the opportunity to combine MIS approaches with a bone conserving technology.

In 1985, Morrey et al. [2] introduced a 60 mm short, double tapered titanium alloy stem with metaphyseal four point fixation, termed the Mayo conservative hip (Zimmer International Inc., Warsaw, Indiana, USA). The Mayo conservative hip is a bone conserving femoral implant, which preserves bone stock at the neck and calcar femoris junction for later revision surgery [6–10]. Although there are several different conservative implants available, only few mid-term results have so far been published [11] and there is only little known about the problems surgeons face when introducing this system into their clinical practise. Therefore, the aim of this study was to evaluate the mid-term outcomes of the first patients treated with the Mayo conservative hip in our University Level 1 department.

## Methods

### Study design

This retrospective cohort study was performed at the Department of Orthopaedic Surgery, University of Wuerzburg, Germany and approved by the local institutional review board (IRB) of the University of Wuerzburg (Nr: 2016020501).

### Setting and participants

Forty-one THAs using the mayo conservative hip were performed between April 2007 and May 2009 in 37 patients (16 women and 21 men). All patients were treated at the Department of Orthopaedic Surgery, University of Wuerzburg, Germany. The mean follow up period was 35 (range 9 - 45) months. The mean age at the time of the surgical intervention was 46.3 (range 16 - 63) years. The indications for THA were primary OA (12 cases, 29.2%), or secondary OA due to developmental dysplasia of the hip (18 cases, 43.9%), femoral head necrosis (6 cases, 14.6%), slippage of femoral epiphysis (4 cases, 9.8%) and M. Perthes (1 case, 2.4%).

Patients with previous operations at the pelvis or proximal femur (e. g. osteotomies, fracture fixation), morbid obesity, metabolic bone disorders and meta-epiphyseal dysplasias were excluded.

### Surgical approach

In 29 cases the transgluteal standard lateral approach was used (Bauer), and in 12 patients the minimally-invasive direct anterior approach (DAA). The surgical techniques used have been described previously by our group [12–15].

### Functional assessment

Functional assessment was performed using the Harris Hip Score (HHS) and the German Extra Short Musculoskeletal Function Assessment Questionnaire (XSFMA-D). All peri- and postoperative complications were recorded. The scores were assessed before and after an average of 35 months following surgery. The XCMFA-D [16], a score based on the Short Musculoskeletal Function Assessment Questionnaire (SMFA), was used to evaluate musculoskeletal function from a patient’s perspective [17]. The XSMFA-D is a 16-item version for routine assessment of functional capacity in patients with orthopaedic disorders. It analyses functional deficits and impairments due to inflammatory, degenerative and injury related causes [17].

### Radiographic analysis

**Fig. 1:**
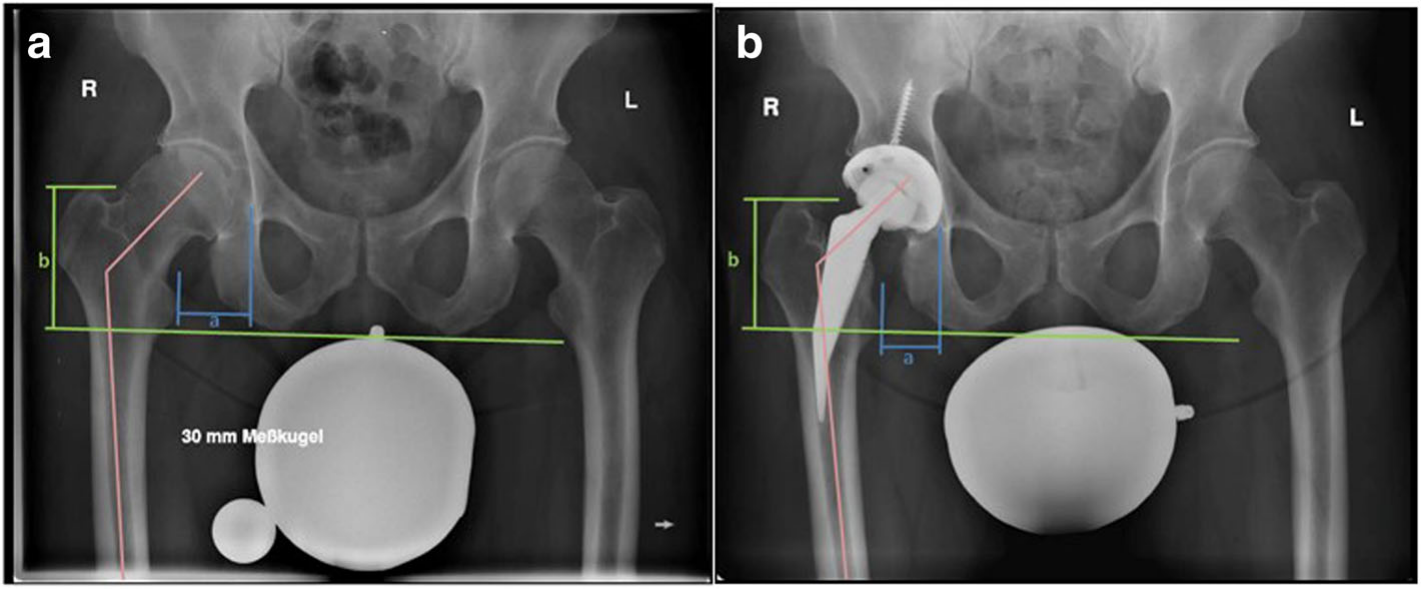
Measurement of lateral offset from the lateral wall of the Koehler’s tear drop of the pelvis and the medial point of the trochanter minor (see measurement **a** within Fig. 1a and b). The leg length (=vertical offset) was measured from the highest point of the trochanter major of the femur to the lowest point of os ischium of the pelvis (see measurement **b** within Fig. 1a and b). The centrum-collum-diaphyseal-angle (see pink line within Fig. 1a and b) was defined preoperatively as the angle between the shaft axis and the femoral neck, and postoperatively the angle between the shaft axis and the neck of the prosthesis

Radiographic analyses were performed using standing anterior-posterior (AP) radiographs of the pelvis and lateral radiographs of the proximal femur pre-operatively, postoperatively and during follow-up. All data were collected in a blinded fashion and random order. The lateral offset was measured between the lateral wall of the Koehler’s tear drop and the medial point of the trochanter minor, while the vertical offset (=leg length) was measured from the highest point of the trochanter major to the lowest point of the os ischium (Fig. 1).

**Fig. 2:**
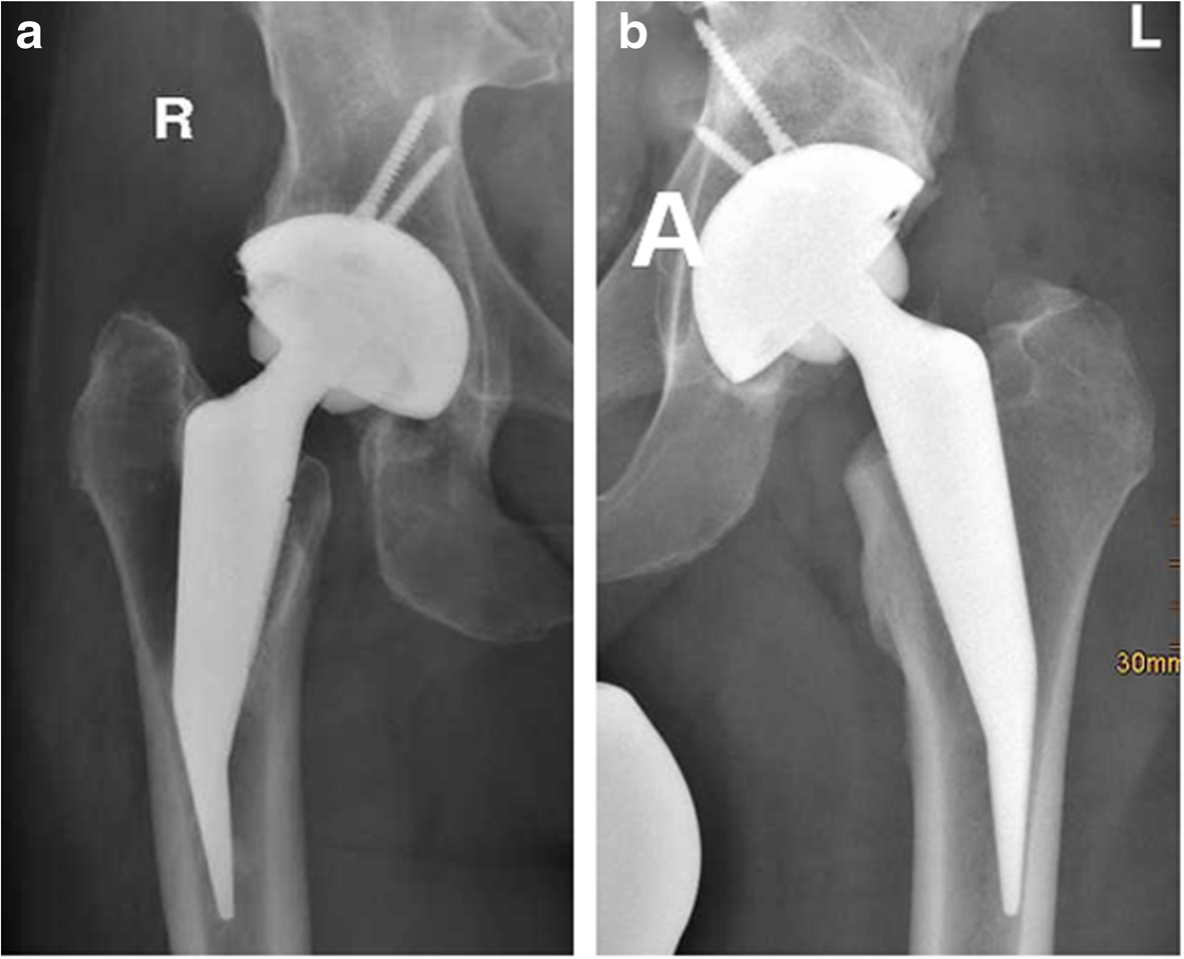
Varus and valgus- alignement of the stem

Stem alignment was measured on postoperative radiographs and classified into three groups: Varus alignment (CCD angle < 120°), neutral alignment (CCD angle 120°-140°) and valgus alignment (CCD angle >140°) (Fig. 2).

The CCD angle was defined pre-operatively as the angle between the shaft axis and the femoral neck, and post-operatively between the shaft axis and the neck of the prosthesis (Fig. 1). The post-operative radiographs were also examined for radiolucency lines, osteolysis or increased density in the periprosthetic zones described by Gruen [10].

### Statistics

Values were given as means, range and SD. We used the software SAS (Version 9.2, SAS Inst. Inc. Cary NC, USA) for all statistical analyses. Chi square analysis was used to compare differences between patients with or without risk factors (BMI, diabetes mellitus, approach). A *p* value of <0.05 was considered significant.

## Results

### Patient characteristics

In five cases an intraoperative non-displaced proximal femoral fracture in the cortex was observed. In four of these patients a lateral approach was used, and one case an anterior approach. Patients with non-displaced proximal femoral fractures exhibited a significantly increased body mass index (27.9 ± 1.9 versus (vs) 23.6 ± 3.6; *p* < .01) and they were significantly older (62.1 ± 8.1 years vs 49.5 ± 8.3 years; *p* < .01) when compared to patients without fractures. Furthermore, the prevalence of diabetes mellitus was significantly higher (*p* = .001) in the patient group with proximal femoral fractures. The usage of a standard lateral approach was not associated with a higher rate of non-displaced proximal femoral fractures when compared to the minimally-invasive anterior approach (*p* = .063) (Table 1).

**Table 1:** Description of the cohort, and comparison between patients characteristics and patients with an intraoperative non-displaced proximal femoral fracture in cortex

Characteristics	All Patients	Intraoperative non-displaced proximal femoral fracture (Type-IA, Vancouver Classification)	*p* value
Age (in years)	49.5 ± 8.3	62.1 ± 8.1	< .01
Diabetes	10/37	4/5	= .001
Body mass index (kg/m^2^)	23.6 ± 3.6	27.9 ± 1.9	< .01
Approach (Bauer)	29/41	4/5	=.063
Developmental dysplasia of the hip	18/41	4/5	=0.03

### Patient reported outcome and complications

**Fig. 3:**
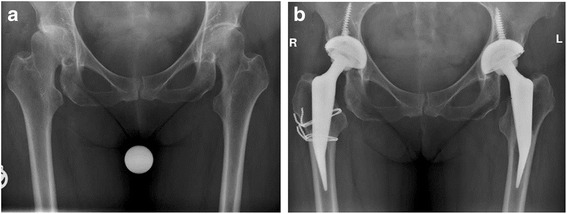
**a** Radiographs of a 49 year old patient with developmental dysplasia of the hip on both sides (**b**) Postoperative view after implantation of a Mayo conservative hip and a Harris Galante II cup (Zimmer). An undisplaced proximal femoral fracture occurred intraoperatively on the right side and was secured with 2 cerclage wires. A significant increased densitiy can be seen in zone 3

The HHS increased from 61.2 (± 7.1) preoperatively to 85.9 (± 6.4) after 35 months, with a range 65 to 91.5. The XSFMA decreased from 30.2 (± 5.2) preoperatively to 12.2 (± 4.3) postoperatively. Complications included intraoperative non-displaced proximal femoral fractures (5 cases, 12.1%, Fig. 3), aseptic loosening (2 cases, 4.9%), acute late onset infection (1 case, 2.4%), insufficient gluteus medius and minimus muscle-function (1 case, 2.4%) and 1 delayed healing (1 case, 2.4%). Implant survival was 95.2% after a mean of 35 months.

In the patient cohort treated with the lateral approach 8 complications were observed, while in the group with the anterior approach 2 complications occured (Table 2). One acute late onset infection 13 months after implantation was treated with exchange of the mobile parts in a one-step procedure.

**Table 2:** Complications related to the surgical approach

Complications	Lateral approach (Bauer)	Anterior approach (DAA)
acute late onset infection	1	
aseptic loosening	1	
delayed wound healing		1
insufficiency glutei muscle	1	
intraoperative non-displaced proximal femoral fracture	4	1
postoperative stem sintering	1	

**Fig. 4:**
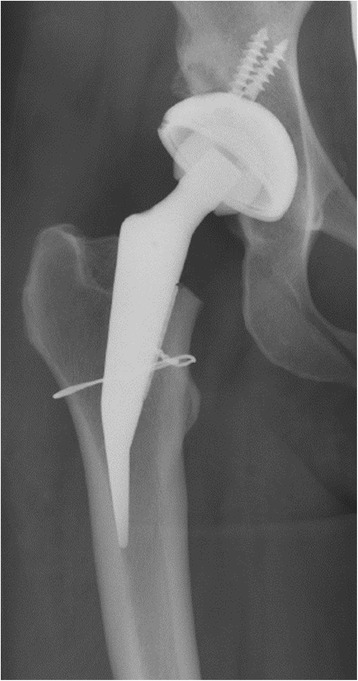
Non displaced periprosthetic fracture of the proximal femur treated with a cerclage

Furthermore, two exchanges of the mayo short stem to a standard taper stem were necessary; One due to aseptic loosening after 13 months, and one due to postoperative stem sintering 13 days following surgery. An intraoperative non-displaced proximal femoral fracture in the cortex was observed in 4 cases in the group with the lateral approach and one case in the group with the anterior approach. Patients with proximal femur fractures were all intraoperative treated with a cerclage to secure the fracture (Fig. 4). Postoperatively, these patients were were limited to non weight bearing for 3 or 6 weeks, and after this 20 kg partial weight bearing before transition to full weight bearing. The occurence of proximal femur fractures did not reach any level of significance related to the performed approach.

### Radiographic analysis

**Fig. 5:**
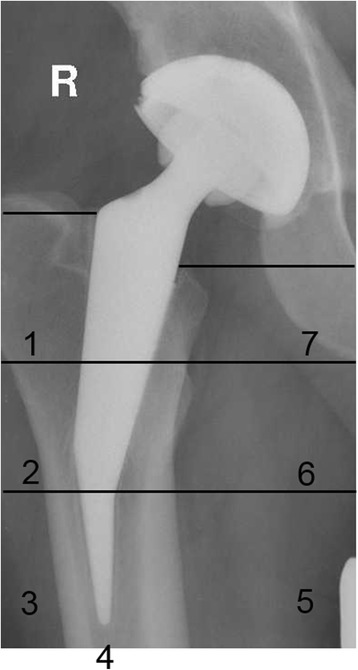
Radiographic appearance of linear radiolucencies in the zones 1, 3 and 5 of Gruen

Radiographic analysis revealed signs of linear radiolucencies in 23 cases in zones 1, 3 and 5 of Gruen (Fig. 5) with the implant reliably fixed.

The CCD angle changed from 134.7° ± 6.4° preoperatively to 132.8° ± 16.9° postoperatively (Table 3). Neutral alignment was observed in 20 (48.7%) hips, varus alignment in 9 (21.9%) and valgus alignment in 12 (29.2%) hips.

**Table 3:** Comparison of preoperative and postoperative lateral offset, leg length and CCD angle

	Preoperative	Postoperative	change
Lateral offset [mm]	43.1 (±7.0)	46.3 (±16.5)	3.9 (±18.5)
leg length [mm]	−2.1 (± 9.9)	2.3 (±11.3)	4.3 (±12.8)
CCD angle	134.7° (±6.4°)	132.8° (± 16.9)	-2.3ˆ (± 18.0)

**Fig. 6:**
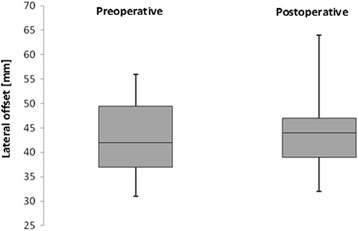
There was no significant change in the pre- and postoperative lateral offset after total hip replacement using the mayo conservative hip

The lateral offset increased from 43.1 ± 7.0 mm preoperatively to 46.2 ± 16.5 mm postoperatively (Fig. 6, Table 3). A correlation between stem alignment and the appearance of periprosthetic fractures was not observed.

**Fig. 7:**
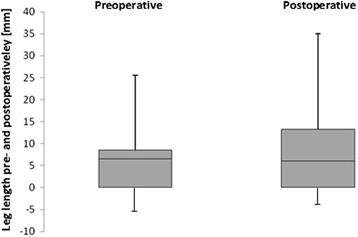
Leg length increased from preoperatively −2,06 mm to postoperatively 2,64 mm

Leg length (=vertical offset) was changed on average by 4.3 ± 12.8 mm (Fig. 7, Table 3).

## Discussion

The mayo conservative hip is a cementless implant with intertrochanteric fixation and preservation of bone stock at the femoral neck and calcar region. Compared to conventionally designed long-stemmed implants, the overall bone resorption over time in these regions is decreased [5, 18].

Preservation of bone stock in younger patients requiring hip replacement is essential since those patients will most likely experience at least one implant revision during their remaining lifetime [1–3]. In our study, HHS improved from 61.2 to 85.6. This can be regarded as a good result (the score is reported as 90-100 for excellent results, 80-90 being good, 70-79 fair, 60-69 poor, and below 60 a failed result) [1, 2, 6]. In a retrospective study, Morrey et al. reported long term results of 159 patients with a mean follow up of 6.2 years.

The survivorship of the Mayo stem was 98.2 after 5 and 10 years. These results are in agreement with the results described by Falez et al. [6], who reported a survivorship of 97.5% after a mean follow up of 4.7 years (range, 1–7 years) a complication rate of 4.3%. In our study, implant survivorship was 95.2% at the 35 months follow-up. This is in line with data for cementless implants provided by the Australian national joint replacement registry [19], describing implant survival rates for patients younger than 65 years of 96-97%.

In our study, the Harris-hip-score (HHS) improved from 66.2 to 85.6. The major intra-operative complication was the occurrence of a non-displaced proximal femoral fracture in the calcar region in 12.1% of the cases. Morrey et al. [1] reported a frequency of 7% whereas studies by Hube et al. [5] and Falez et al. [6] reported a frequency of 2.2 and 2.5%, respectively.

The higher complication rate in our group can be attributed to the surgeons’ learning curves. The more obese patient cohort in our study compared to previously reported outcome analyses may be another reason for the relatively high complication rate. Due to acute late onset infection 13 months after implantation, one patient was treated by revision of the mobile implant parts in a one-step procedure. Other revisions were not necessary.

The Swedish hip arthroplasty registry [20] reported 5 year revision rates for patients under 60 years being 2% excluding interventions to replace acetabular components due to primary instabilities. Survival rates without revision of a femoral short-stem component and aseptic loosening have been reported as 98% after 6.8 years in 162 treatments [1], 99% in 155 cases after 6.2 years [21], 100% after 5.2 years [22] and 95, 2% in the current study.

The fractures of this study occurred during stem insertion. In that case, the stem was removed, cerclage-wires were placed around the minor trochanter of the femur and re-insertion of the stem was performed (Fig.4). Afterwards, patients were treated without weight bearing for 3 or 6 weeks, and after this 20 kg partial weight bearing before transition to full weight bearing. Another recent study showed that patients treated similarly to ours after intraoperative fracture using a mayo short stem prosthesis, had no disadvantages referring stem migration or alignment [23].

We assumed that the fractures occurred due to high radial tension evolving in the lumen of the femur during stem insertion [24]. Previous studies [9, 10, 22, 24] found that the fissures have mostly been located at the point where the cortex is thinnest and therefore most vulnerable, as it was in the current study. The fractures are almost always found to be parallel to the metaphyseal axis. Moreover, they have been found to be usually not detectable in conventional radiographic imaging [24]. This is due to the fact that longitudinal fractures often coincide with the projection of the stem.

Fractures that are located medially remain often clinically undetected and the implant is therefore not sufficiently fixed. Within this context, diminished bone density has been described as predisposing factor for periprosthetic fractures [6, 24, 25]. Another study including a low number of patients treated with the mayo stem also showed increased numbers of intraoperative fractures (13.3%) with 2 cases in 15 patients [26]. In the present study, diabetes, higher body mass index and older ages are shown to be risk factors for intraoperative periprosthetic fracture (*p* < 0.01).

Good functional results and good short-term and mid-term survival rates have been reported for various short-stem hip designs [6, 10, 19, 27]. An advantage of SHA is the potential preservation of the femoral bone stock making it possible to use of a conventional stem if a revision should become necessary, thus avoiding revision implants [6, 24]. Furthermore, advantages of SHA include a reduced soft-tissue trauma and a more physiological load transfer at the metaphyseal part of the femur. The curved small stem designs facilitate the preparation and the insertion of the stem [6] resulting in reduced hospital length of stay and a faster post-operative mobilisation [24].

In summary, literature is conflicting with overall cumulative failure rates between 0.9 and 5.7% [28], which is in line with the results of this study with a failure rate of 4.8%. Overall, SHA offers a sufficient alternative, especially for younger patients requiring hip replacement. However, surgeons should be aware of potential risk factors such as diabetes mellitus and/or obesity, especially when they start to use the described short stem system.

## Conclusion

In this cohort, THA with the Mayo conservative hip shows acceptable mid-term results with an improvement of the HHS from 61.2 to 85.6. A higher complication rate compared to conventional cementless designs was observed, with a high rate of non-displaced proximal femoral fractures. This could be attributed to the surgeon’s learning curve, the stem design and patient inherent risk factors such as diabetes mellitus and/or obesity. Therefore surgeons using this implant should be aware of the inherent flat learning curve of the system and be particularly careful in their patient selection. Intraoperative fluoroscopic imaging is highly recommendedto detect periprosthetic femoral fissures.

### Limitations

Only short-term and mid-term results are available for SHA and these results still have to be confirmed by long-term studies and larger patient cohorts with a focus on risk factors.

## Abbreviations

AP: Anterior-posterior
BMI: Body mass index
DAA: Direct anterior approach
HHS: Harris Hip Score
MIS: Minimal invasive surgery
OA: Osteoarthritis
SMFA: Short Musculoskeletal Function Assessment Questionnaire
THA: Total hip arthroplasty
XSFMA-D: German Extra Short Musculoskeletal Function Assessment Questionnaire
